# Utilization of artificial intelligence approach for prediction of DLP values for abdominal CT scans: A high accuracy estimation for risk assessment

**DOI:** 10.3389/fpubh.2022.892789

**Published:** 2022-07-28

**Authors:** H. O. Tekin, Faisal Almisned, T. T. Erguzel, Mohamed M. Abuzaid, W. Elshami, Antoaneta Ene, Shams A. M. Issa, Hesham M. H. Zakaly

**Affiliations:** ^1^Department of Medical Diagnostic Imaging, College of Health Sciences, University of Sharjah, Sharjah, United Arab Emirates; ^2^Computer Engineering Department, Faculty of Engineering and Natural Sciences, Istinye University, Istanbul, Turkey; ^3^Department Information Systems, College of Computer and Information Sciences, King Saud University, Riyadh, Saudi Arabia; ^4^Department of Software Engineering, Faculty of Engineering and Natural Sciences, Uskudar University, Istanbul, Turkey; ^5^Department of Chemistry, Physics and Environment, Faculty of Sciences and Environment, INPOLDE Research Center, Dunarea de Jos University of Galati, Galati, Romania; ^6^Physics Department, Faculty of Science, Al-Azhar University, Assiut, Egypt; ^7^Physics Department, Faculty of Science, University of Tabuk, Tabuk, Saudi Arabia; ^8^Experimental Physics Department, Institute of Physics and Technology, Ural Federal University, Ekaterinburg, Russia

**Keywords:** artificial intelligence (AI), computed tomography, DLP, abdominal, artificial neural network (ANN)

## Abstract

**Purpose:**

This study aimed to evaluate Artificial Neural Network (ANN) modeling to estimate the significant dose length product (DLP) value during the abdominal CT examinations for quality assurance in a retrospective, cross-sectional study.

**Methods:**

The structure of the ANN model was designed considering various input parameters, namely patient weight, patient size, body mass index, mean CTDI volume, scanning length, kVp, mAs, exposure time per rotation, and pitch factor. The aforementioned examination details of 551 abdominal CT scans were used as retrospective data. Different types of learning algorithms such as Levenberg-Marquardt, Bayesian and Scaled-Conjugate Gradient were checked in terms of the accuracy of the training data.

**Results:**

The *R*-value representing the correlation coefficient for the real system and system output is given as 0.925, 0.785, and 0.854 for the Levenberg-Marquardt, Bayesian, and Scaled-Conjugate Gradient algorithms, respectively. The findings showed that the Levenberg-Marquardt algorithm comprehensively detects DLP values for abdominal CT examinations. It can be a helpful approach to simplify CT quality assurance.

**Conclusion:**

It can be concluded that outcomes of this novel artificial intelligence method can be used for high accuracy DLP estimations before the abdominal CT examinations, where the radiation-related risk factors are high or risk evaluation of multiple CT scans is needed for patients in terms of ALARA. Likewise, it can be concluded that artificial learning methods are powerful tools and can be used for different types of radiation-related risk assessments for quality assurance in diagnostic radiology.

## Introduction

The advantages of CT have led to a significant increase in its use as they produce fast image acquisition in high quality. Conversely, the risk of contracting cancer due to absorbed radiation dose during imaging procedures is of major concern to patients of all ages, including children and pregnant women (1, 2). In addition to accurately estimating the patient radiation dosage, it is important to examine the possible absorbed radiation dose during CT examinations for patients. Clinical studies in X-ray radiographic imaging have demonstrated that radiation doses to patients can be significantly reduced without compromising patient diagnosis. It is expected that radiation doses to patients would be reduced by half if the patient's body thickness is decreased by 1 cm, in comparison with current clinical practice, without compromising patient diagnosis. Zheng et al. derived a general equation for the optimal selection of scan parameters in X-ray imaging, including CT and radiographic imaging (3). The radiation dosage the patient receives depends on several factors, including the type of CT system and the acquisition process used. There is a considerable effort in reducing radiation doses to patients whilst maintaining accurate diagnosis in CT imaging worldwide (4–6). Reported evidence was drawn from phantom studies that involve newborns as well as adult participants. Such phantoms have been often used in dosage measurements of the patients' dosimetry in diagnostic radiography (7). Another method for CT dosimetry is known as the Monte Carlo simulation method. The expression “Mathematical phantoms” is used to stand in for the ordinary Monte-Carlo patients. The number of mathematical phantoms has doubled in the previous decade. Additional scanners and patient-specific modeling models have been employed to arrive at an accurate dosage from CT studies (8). Using an ordinary Monte-Carlo software package to measure individual patient-specific radiation doses could be needed. Monte Carlo simulation is highly effective for dose prediction. It is a time-consuming process and operators must have the knowledge to carry out the CT scanner tests, but they are not necessary to simulate CT scanners (9). artificial neural networks (ANN) offer a flexible approach to aid physicians and researchers in dealing with large amounts of complex medical data (10–15). The literature verifies that some previous studies were performed in terms of utilization of Artificial Intelligence (AI) in diagnostic radiology. Meineke et al. used a machine-learning algorithm to assess dosage optimality in CT efficiency (16). In another paper, Sinha et al. studied the use of artificial neural networks in pediatric radiographs with regard to their potential in the identification of CT findings (17). They found that the ANN model is more sensitive (82.2%) than physician estimation (62.2%). Their results showed that with dosage optimization, machine learning (ML) would comprehensively and rapidly detect CT scans (17). It may be used to further automate CT quality assurance. McCollough and Leng investigated the application of artificial intelligence in CT dose optimization (18). Their results showed that AI-based techniques that automate the organ and pathologic characterization had been safely transported from testing to clinical practice, boosting the justification for medical CT scanning on the patient's side. To the best of our knowledge, DLP estimation using 10 different scan-related input parameters for Artificial Neural networks (ANN) for abdominal CT scans was not performed in the literature. Therefore, we sharply focused on generating a novel dose-length-product (DLP) estimation algorithm using advanced artificial learning methods. The Artificial Neural networks (ANN) are used to estimate the DLP compared to the ordinary methods based on real patients' parameters and the results are compared and validated. We proposed that Artificial neural networks (ANN) can assist in CT quality assurance in identifying optimal doses and have already been missed by current quality assurance methods. The authors believe that the integration of outcomes will improve the knowledge gap in DLP estimation when patients and protocol parameters are known and complete. Although there is a close relationship between CTDI and DLP values, technological issues and software mistakes that arise in DLP values in everyday clinical practice interrupt scientific investigations and create some difficulty in understanding. The purpose of this research is to assess the DLP values that will be utilized during the various studies, particularly across hospitals, in light of the algorithm produced by evaluating the reliability. Thus, DLF values for data sets collected from different centers may be recalculated using this approach, and any quantitative disparities can be highlighted to offer a better understanding of the present technical challenge.

## Materials and methods

### Study design

In the current study, we developed a DLP estimation method involving the use of procedure- parameters and patient characteristics. AI approaches using MATLAB® neural fitting (NF) tool were utilized for Artificial neural networks (ANN) modeling of DLP estimation algorithm. Abdominal CT scans were implemented on *GE Optima 660*, 128 slice-CT scanners. A cumulative number of *N* = 551 abdominal scans along with their information are used for testing and training the Artificial neural networks (ANN) algorithm. To reduce the error in the model output and the mean square error was used as the cost function, provided in Equation (1).


(1) $$Mean\;Square\;Error\;(MSE)\;=\;1/\;n(\sum_{1}^{n}{\left(Y_{i}-{Y^{\prime }}_{i}\right)}^{2}$$

Where; n is the number of data points, Y_i_ is observed values, and Y'_i_ is the predicted value.

The structure of the ANN model was designed considering various input parameters such as patient weight, patient size, body mass index, mean CT volume index volume (CTDI), scanning length, kVp, mAs, and exposure time per rotation, and pitch factor. The aforementioned examination details of 551 abdominal CT scans were used as retrospective data without any patient information. Different types of learning algorithms such as Levenberg-Marquardt, Bayesian and Scaled-Conjugate Gradient were checked in terms of the accuracy of training data.

### Back propagation neural networks

Artificial Neural Network is used for solving problems in analyzing data with high resolution and non-linear characteristics like biomedical signals, with its good generalization, high predictive ability, and the ability to learn complex and non-linear relations of input and output (19–21). For ANNs, the practice of doing as well as the observed result is much more important than the use of laws. An indication of probable results that is unrelated to human fatigue and which uses high-prediction ability is useful in evaluating conditions such as these. Similarly, ANNs are capable of rapid detection, estimation, rapid classifications, and continuous study of analysis. This study used the Back Propagation (BP) algorithm to teach learners how to classify commonly used patterns. Although the various forms and designs of neural networks differ significantly in how they learn, these characteristics are well-recorded in the literature. A BPNN is a multilayer feed-forward network the BP method applies a distributed computing model to treat both qualitative and quantitative data. It has high responsiveness, robustness, and adaptability and can perfectly simulate any non-linear relationship. Therefore, BPNNs works well for dense and high-quality medical data, which can be noisy and potentially unreliable. The network's architecture is layered, which is often referred to as a feed-forward, and the information flows from the input layer to the output layer through the hidden layer (s).

## Results

**Figure 1:**
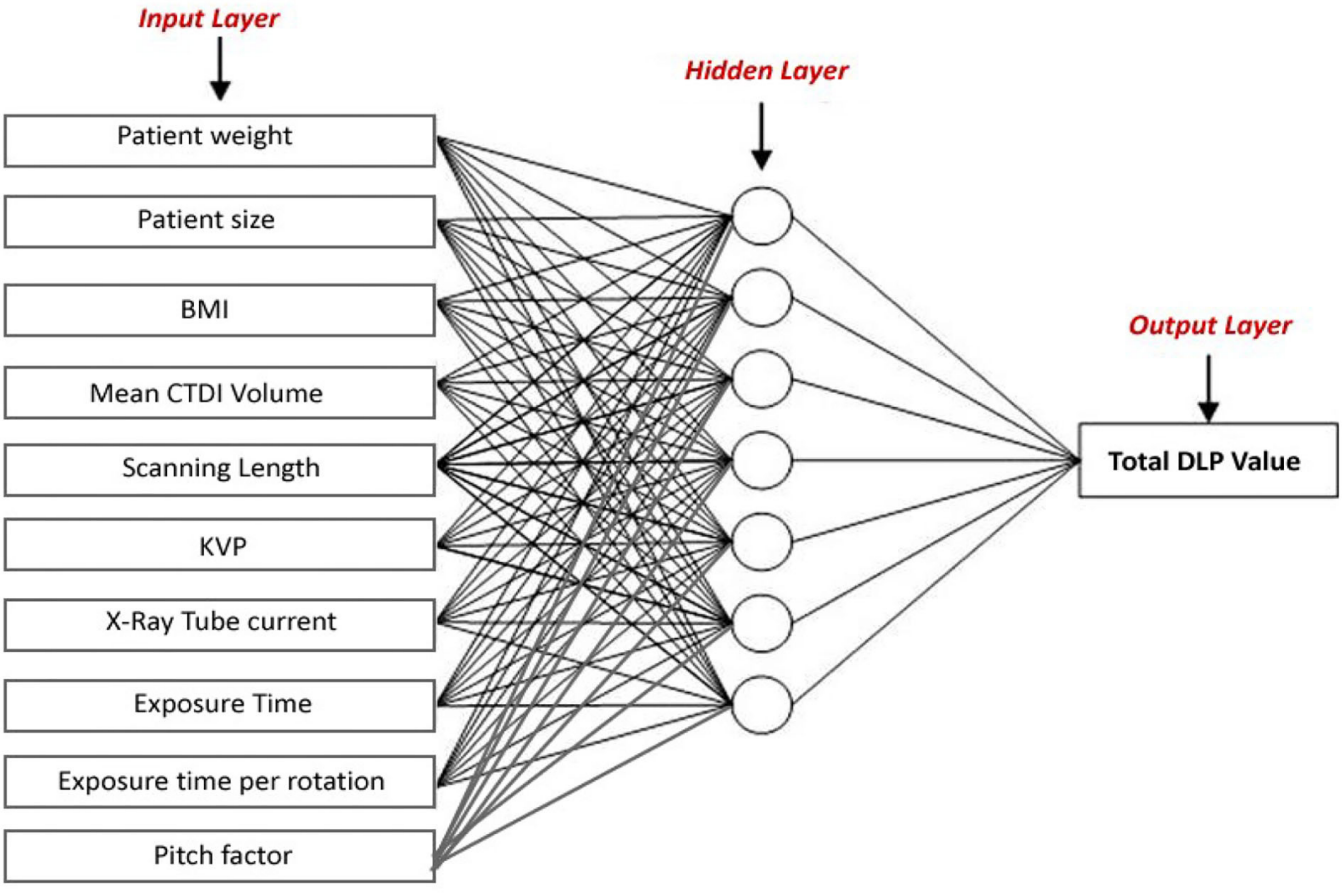
The structure of the generated ANN model.

**Figure 2:**
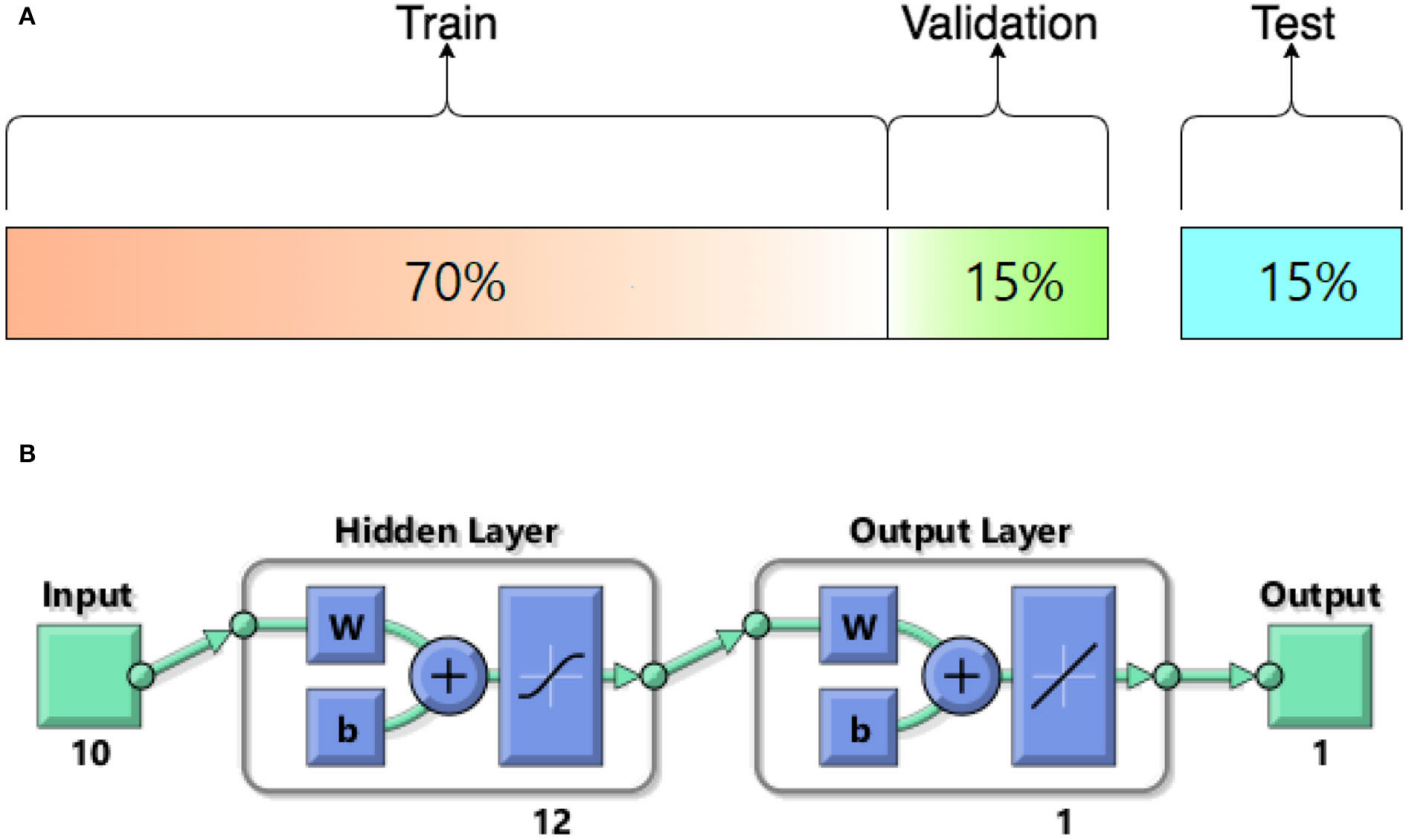
**(A)** The structure of modeled learning algorithm in MATLAB-NF tool **(B)** Numerical ratios of the train, validation, and test data as a percentage of *N* = 551.

**Figure 3:**
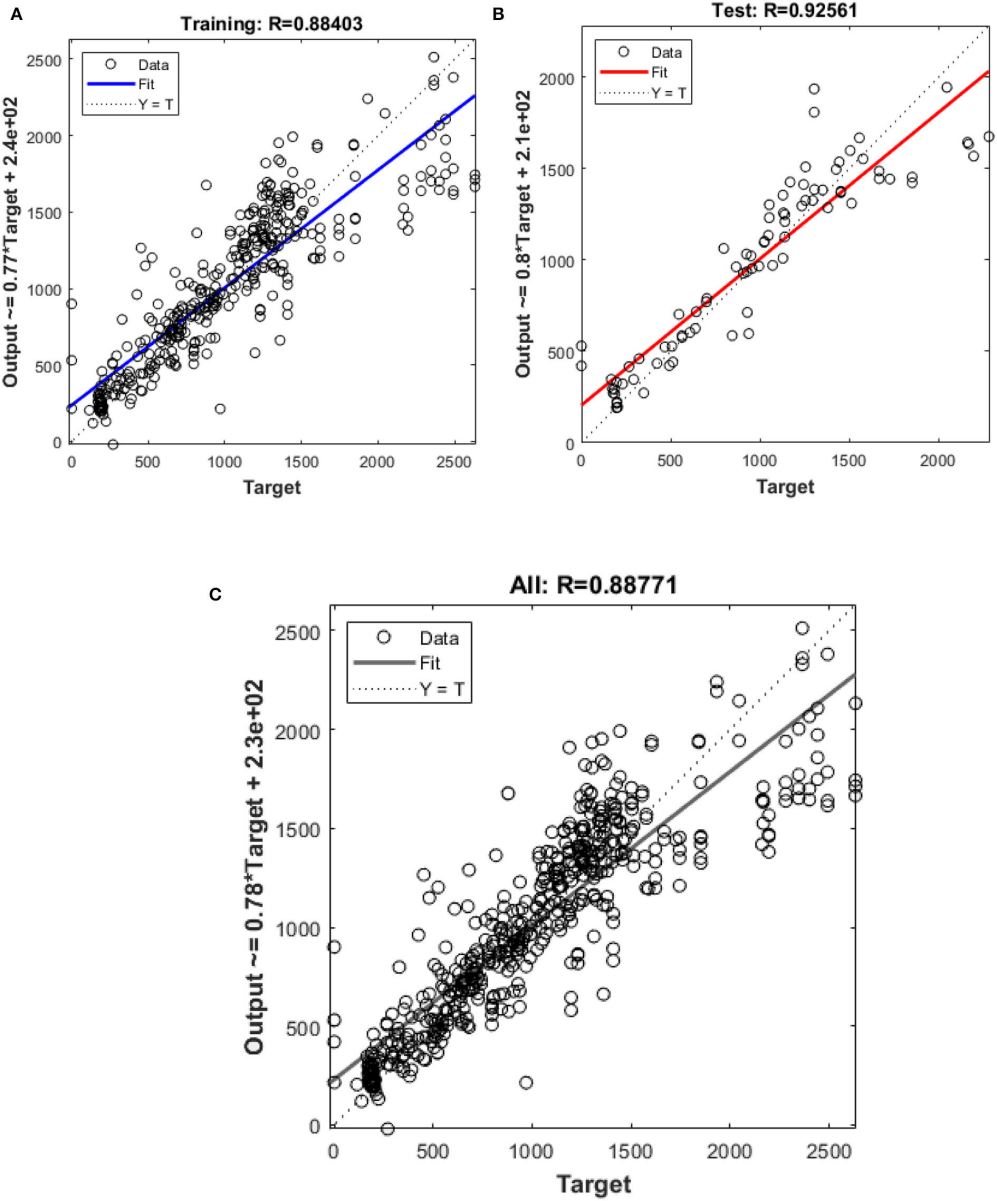
Outputs of **(A)** training **(B)** test **(C)** all data sets by Levenberg-Marquardt algorithm as a function of actual (target) DLP.

**Figure 4:**
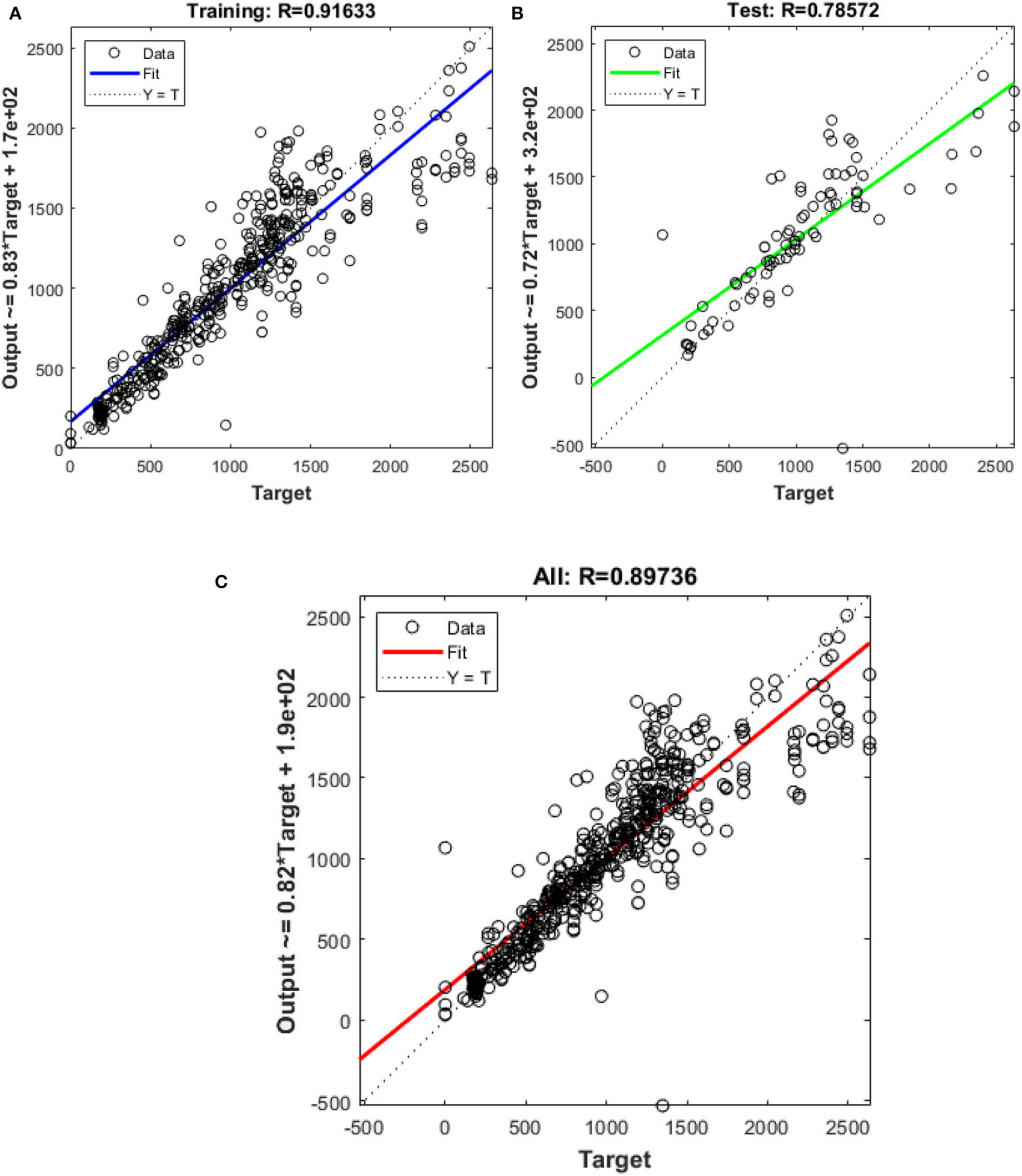
Outputs of **(A)** training **(B)** test **(C)** all data sets by Bayesian algorithm as a function of actual (target) DLP.

**Figure 5:**
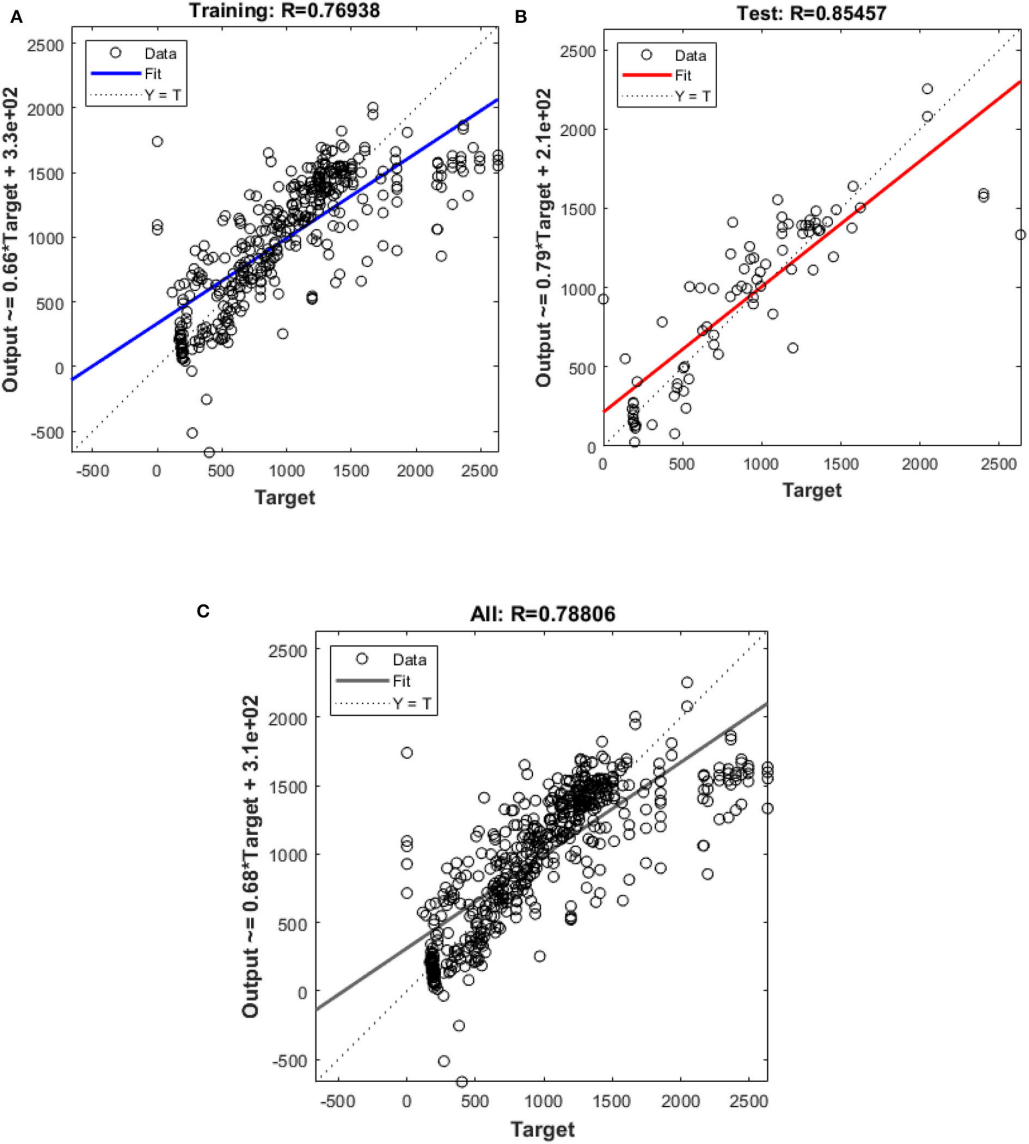
Outputs of **(A)** Training and **(B)** test **(C)** all data sets by Scaled-Conjugate Gradient algorithm as a function of actual (target) DLP.

**Figure 6:**
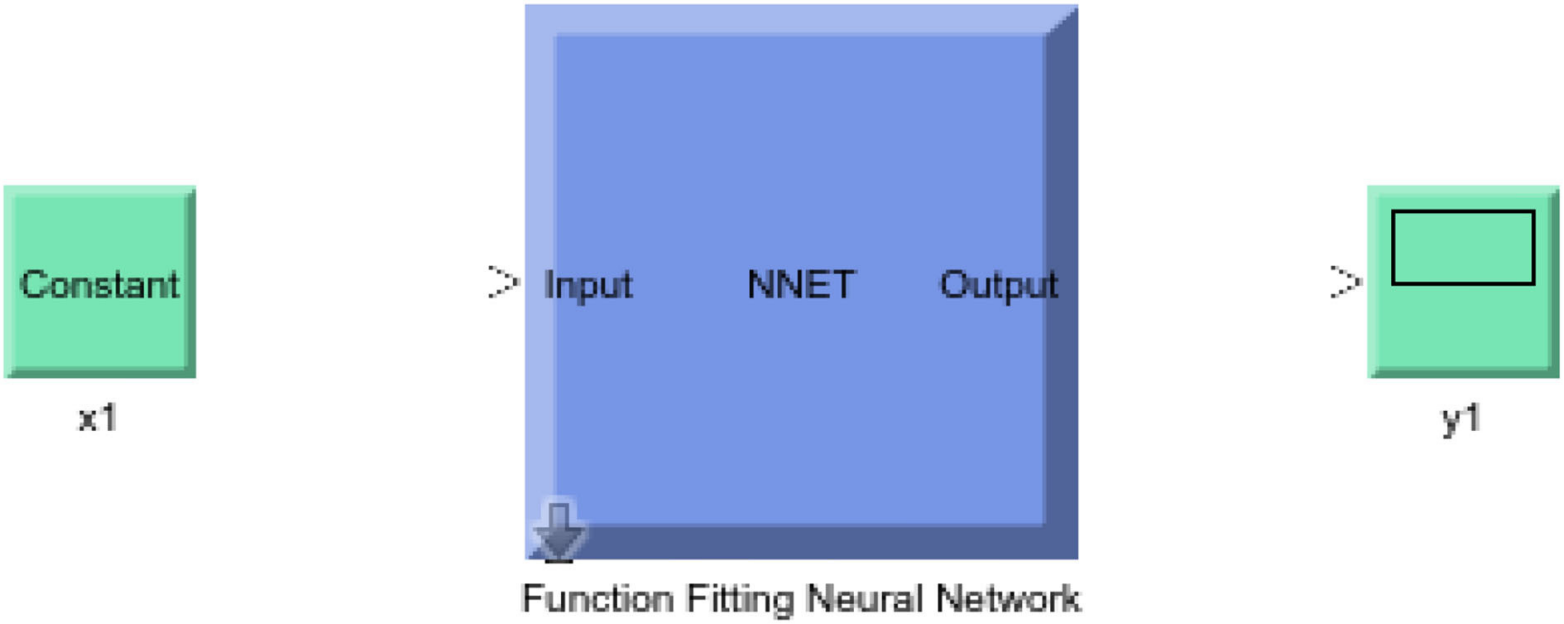
Obtained Simulink by Levenberg-Marquardt algorithm.

In this study, different types of learning algorithms such as Levenberg-Marquardt, Bayesian and Scaled-Conjugate Gradient were checked in terms of accuracy of training data. We considered the same amount of training, validation, and test data sets as 70, 15, and 30%, respectively. Figure 1 shows the structure of the generated ANN model along with the information on input layer parameters. Input data are received from 551 patients with 10 features namely patient weight (kg), patient size (cm), BMI, mean CTDI volume, scanning length, KVP, X-ray tube current, exposure time, exposure time per rotation, and pitch factor, respectively. A randomized selection has been used for 385 (***70%** of N*) training data, 83 (***15%** of N*) validation data, and 83 (***15%** of N*) testing data (see Figure 2A). In this study, twelve neurons were utilized in the hidden layer of the learning algorithm. Accordingly, we used a sigmoid-transfer function in each neuron due to its non-linear behaviors. The structure of the modeled learning algorithm in MATLAB-NF can be observed in Figure 2B. The learning factor was set to 0.04 and the momentum factor to 0.1. Figures 3A, 4A, 5A show the outputs of training data sets as a function of actual (target) DLP obtained by the Levenberg-Marquardt algorithm, Bayesian algorithm, and Scaled-Conjugate Gradient algorithm, respectively. Using training data in ANN programs is a straightforward term, but it is at the heart of these techniques. They imitate the human brain's ability to weigh complex stimuli and help produce corresponding decisions in the neurons. In this study, basic training data were used to assist the algorithm in learning how to implement neural network technology to estimate DLP values (output). We reported the *R*-values of training data as 0.88403, 0.91633, and 0.76938 for the Levenberg-Marquardt algorithm, Bayesian algorithm, and Scaled-Conjugate Gradient algorithm, respectively. The maximum *R*-value for the overall training process (*R* = 0.91633) was reported for the Bayesian algorithm. On the other hand, we used test data to check the learning process after it has been set to train on an initial dataset. The theory is that predictive models will still need to be confirmed by use. The test data is the only algorithm that checks the ANN algorithm's overall precision in a purely quantitative manner. In this study, the test data collection from the Levenberg-Marquardt algorithm, Bayesian algorithm, and Scaled-Conjugate Gradient algorithm is also used for the ANN model validation. The meaning of test data in AI applications is highly important since the abilities of a designed algorithm can be observed during the testing of prediction as a function of existing values. Figures 3B, 4B, 5B depict the correlation of test data as a function of a target value. We reported the *R*-values of test data as 0.92561, 0.78572, and 0.85457 for the Levenberg-Marquardt algorithm, Bayesian algorithm, and Scaled-Conjugate Gradient algorithm, respectively. The maximum R-value for the overall training process (*R* = 0.92561) was reported for the Levenberg-Marquardt algorithm, which provided an equation for the **output** as 0.8^*^Targe + 2.1e+02, where the target is the real and observed value. Finally, fifty independent examinations were checked in terms of accuracy in the obtained learning algorithm in Levenberg-Marquardt. However, it‘s worth mentioning that the correlation of all learning algorithms was reported above 0.78 (see Figures 3C, 4C, 5C). Figure 6 shows the obtained Simulink by the Levenberg-Marquardt algorithm. The correlation of the Levenberg-Marquardt algorithm for all data was also reported as *R* = 0.88771. The ten different input parameters were defined in the constant x1 box of obtained Simulink. The obtained results showed that the provided algorithm is able to estimate DLP values with high accuracy. As a result of this study, it can be said that the Levenberg-Marquard-based learning algorithm, which was created with 12 hidden layers and 551 abdominal CT scan data (each exam contains 10 different scan parameters), provides the highest level of prediction rate at 0.92561.

## Discussions

This study showed that it is possible to do a large-scale CT dose analysis for DLP values in abdominal CT scans using the ANN model. This is a comprehensive study that shows the importance of AI methods when applied to a large number of CT exams with their meaningful inputs and accordingly output. It is well-known fact that the recommended CT dosage is calculated as dose index (CTDIvol) or dose length product (DLP) (22, 23). Therefore, the main output was considered DLP value, whereas the potential parameters that can affect the DLP values were patient weight, patient size, body mass index, mean CTDI volume, scanning length, kVp mAs, and exposure time per rotation, and pitch factor. The study of how the brain operates can also lend itself to using neural networks, as the layers in the networks mimic the various processing stages of the brain and how it uses data can be studied in almost the same manner. A neuron is connected to the layer above it. The single-layer method results are computed as the product of each input unit being joined with the next using a linear operation, which connects these two neurons in a row in the hidden layer, e.g., with non-linear activation functions, when in the regression model. It has been found that network-based regression models can yield better results than the latter, as a result of a discovery of wider use. We conducted ANN to evaluate the possibility of using DLP prediction on a wide sample as part of CT assurance for results. The term of dose assessment for risky patients has long been a significant concern and numerous systems for managing community doses have been developed. Since the AI used for CT dose optimization involves the use of several scan parameters, we believe that this approach is an even larger number of scans. The literature review showed that many novel studies were performed in terms of the utilization of AI in different sub-areas of radiological sciences (16, 17, 24–28). However, to our understanding, no study has attempted to examine the usefulness of the ANN algorithm with comprehensive learning models in abdominal CT scans yet. Therefore, our study provided not only a high accuracy prediction on DLP values but also evaluated different learning algorithms such as Levenberg-Marquardt, Bayesian and Scaled-Conjugate Gradient, comparatively.

The main limitation of our investigation was to perform retrospective analysis, but this is very important when assessing radiation dose as it is impossible to experiment on humans. However, a large number of CT scans, along with their input parameters, enabled the neural networks to work with high efficiency. In clinical routine, DLP is a value that can be obtained as a result of a CT scan. It means that pre-assessment is physically challenging for patients in terms of risks from ionizing radiation. Since we obtained a high accuracy DLP estimation in our novel algorithm, one can say that a risky patient can be evaluated before a potential abdominal CT scan. The current investigation obtained a highly accurate DLP estimation that can be used to assess the radiation dose for the risky patient before an abdominal CT scan. Absorbed x-ray radiation can destroy molecules and introduce new mutations. A high level of radiation damages human tissue, as shown by the degree of skin burns and the likelihood of cancer. Since a higher dosage is often associated with cancer, it is therefore expected that a lower dose would be, too (29, 30). There is actually no direct research evidence that shows that low radiation levels (such as those used in imaging) have an increased risk of cancer (31, 32). Therefore, any type of risk assessment prior to CT examination would be beneficial for patients in terms of the ALARA principle. The outcomes of the recent investigation can be considered as a practical tool and reflection of promising artificial intelligence methods for diagnostic radiology routines. Moreover, this type of practical approach can increase the awareness of radiographers on artificial intelligence in addition to dose reduction strategies, while they are in an adaptation process on involvement in the future's radiology (33, 34). In summary, ANN will comprehensively identify DLP values in abdominal CT scans that could benefit from dose optimization. It can help streamline the process of CT quality assurance, making dose data more extensive and less time consuming.

## Conclusion

In this retrospective, cross-sectional study, the goal was to test Artificial Neural Network (ANN) modeling to estimate the significant dose length product (DLP) value during abdomen CT scans for quality assurance. The ANN model was built with several input factors in mind, including patient weight, patient size, BMI, mean CTDI volume, scanning length, kVp, mAs, exposure time per rotation, and pitch factor. As retrospective data, the aforementioned examination details of 551 abdominal CT scans were employed. The correctness of the training data was tested using several learning methods such as Levenberg-Marquardt, Bayesian, and Scaled-Conjugate Gradient. For the Levenberg-Marquardt, Bayesian, and Scaled-Conjugate Gradient algorithms, the *R*-value is the correlation coefficient for the real system, and the system output is 0.925, 0.785, and 0.854, respectively. The researchers discovered that the Levenberg-Marquardt algorithm accurately detects DLP levels in abdominal CT scans. It can be a good way to make CT quality assurance easier. It can be concluded that the results of this innovative artificial intelligence system can be employed for high-precision DLP estimations before abdominal CT tests, especially when radiation-related risk variables are high or several CT images are required for ALARA risk evaluation. Similarly, artificial learning approaches may be concluded to be powerful tools that can be employed for many forms of radiation-related risk evaluations for quality assurance in diagnostic radiology.

## Data availability statement

The original contributions presented in the study are included in the article/supplementary material, further inquiries can be directed to the corresponding author/s.

## Author contributions

HT, HZ, SI, and MA: wrote the main manuscript text. WE, FA, TE, and AE: prepared and drown all figures. All authors reviewed and revised the manuscript. All authors have read and agreed to the published version of the manuscript.

## Funding

The article processing charge was funded by “Dunarea de Jos” University of Galati, Romania.

## Conflict of interest

The authors declare that the research was conducted in the absence of any commercial or financial relationships that could be construed as a potential conflict of interest.

## Publisher's note

All claims expressed in this article are solely those of the authors and do not necessarily represent those of their affiliated organizations, or those of the publisher, the editors and the reviewers. Any product that may be evaluated in this article, or claim that may be made by its manufacturer, is not guaranteed or endorsed by the publisher.

## Abbreviations

ANN: Artificial neural networks
BP: Back Propagation
BPNN: Back Propagation Neural Networks
CTDIvol: CT volume index
DLP: dose-length-product
kVp: Kilo Voltage peak
mAs: milli Ampere-seconds
ML: machine learning
MSE: Mean Square Error
NF: neural fitting.
